# Low-grade myofibroblastic sarcomas of the maxilla

**DOI:** 10.3892/ol.2014.2790

**Published:** 2014-12-11

**Authors:** JIN-YU QIU, PENG LIU, CE SHI, BING HAN

**Affiliations:** 1 Department of Oral and Maxillofacial Surgery, Hospital of Stomatology, Jilin University, Changchun, P.R. China; 2 Stomatology Department, School of Medicine, Yanbian University, Yanji, Jilin, P.R. China

**Keywords:** low-grade myofibroblastic sarcoma, inflammatory myofibroblastic tumor, primary lesion, prognosis

## Abstract

Low-grade myofibroblastic sarcoma (LGMS) is a distinct mesenchymal myofibroblastic malignancy. The tumor may occur at a variety of sites, but is particularly associated with the head and neck. Of the two maxillary sarcomas that were analyzed in the present study, one was misdiagnosed as an inflammatory myofibroblastic tumor during pre-operative excision biopsy, and later presented with a different immunophenotype upon recurrence. Representative paraffin blocks from formalin-fixed tissues were selected from each patient and designated as case 1 and case 2. Immunohistochemical studies were performed on 3-μm thick sections using primary antibodies against α-smooth muscle actin (α-SMA), muscle-specific actin (MSA), desmin, vimentin, calponin, h-caldesmon, fibronectin, cytokeratin, cluster of differentiation 34 (CD34), S-100 protein, anaplastic lymphoma kinase (ALK), epithelial membrane antigen (EMA) and Ki-67. Immunohistochemistry was performed using the streptavidin-biotin-peroxidase complex method. The tumor cells from the two maxillary LGMSs, including the recurrent lesion, were positive for vimentin and fibronectin, and negative for S-100 protein, CD34, EMA, h-caldesmon, ALK, MSA and calponin. The tumor cells from case 1 demonstrated positive staining for α-SMA protein and negative staining for desmin. By contrast, the tumor cells from the primary lesion in case 2 presented with negative staining for α-SMA and positive staining for desmin, while the cells of the recurrent lesion were α-SMA-positive and desmin-negative. The present study concluded that cases of LGMS with immunoprofile alterations are predictive of relatively poor prognoses.

## Introduction

Myofibroblasts, initially identified in granulation tissue in 1971 (1), have been revealed as the principal cell type in certain neoplastic soft-tissue lesions, and occasionally in the stroma of normal tissues (2,3). Myofibroblasts have also been revealed to be associated with inflammatory or fibrosing non-neoplastic conditions (2,3). In adults, myofibroblasts are known to exist in the periodontal ligaments and around the testicular seminiferous tubules (4). The cells are believed to originate from proximal resident mesenchymal cells, in particular fibroblasts, and less frequently from smooth muscle cells, pericytes and endothelial cells (5–7). In a previous study, Gabbiani (8) concluded that the transition from fibroblast to myofibroblast resulted from the combined effect of mechanical tension, transforming growth factor-β and extra domain-A of cellular fibronectin (8). However, alternative mechanisms to explain the cellular origin of myofibroblasts have also been proposed; these include the recruitment from bone marrow-derived circulating fibrocytes and the occurrence of epithelial-mesenchymal transformation (9). Evidence to suggest that myofibroblasts can differentiate into smooth muscle cells is yet to be published, and those cells present during wound healing are believed to apoptose following the completion of epithelialization (8,10).

Myofibroblasts are spindle-shaped, bipolar or stellate cells with elongated, tapered, indented or wavy nuclei. The cells may also appear as short, oval and pale-staining cells with distinct and punctate, small, central nucleoli (11). The cytoplasm is usually light eosinophilic or occasionally amphophilic, moderate to minimal in amount and with indistinct cellular margins. Ultrastructurally, the cytoplasm is comprised of abundant rough endoplasmic reticulum, and subplasmalemmal or peripheral bundles of thin cytoplasmic filaments with dense foci. These bundles pass through the cell membrane to form the cell-to-matrix junction with extracellular fibronectin fibrils, a structure known as the fibronexus (12). The fibronexus is observed in cells of reactive, and other forms, of myofibroblastic lesions, but is usually absent in smooth-muscle cells and fibroblasts (13). In contrast to myofibroblasts, myofilaments are distributed throughout the cytoplasm in smooth muscle cells, are accompanied by cell membrane vesicles and plaques, and are surrounded by the external lamina (12).

Low-grade myofibroblastic sarcomas (LGMSs) have been reported in the literature under various terms, including myofibrosarcomas, sarcomas of myofibroblasts, myofibroblast-rich fibrosarcomas, leiomyosarcoma myofibroblastic variant and spindle-cell sarcomas showing myofibroblastic differentiation (14). Following the establishment of a diagnostic criteria by Mentzel *et al* (15) in 1998, LGMS was reclassified as a distinct entity by the World Health Organization classification of soft-tissue tumors (16). LGMSs are primarily composed of spindle-shaped or stellate cells arranged in fascicles of varying length, with or without focal herringbone or storiform whorls (11,12). Tumor cells consist of small to moderate amounts of ill-defined, palely eosinophilic cytoplasm and fusiform nuclei, which may be tapering and wavy, or round and vesicular with indentations and small, indistinct nucleoli. Focal nuclear atypia is observed in the majority of cases, but is usually mild with dispersed, enlarged hyperchromatic nuclei. However, larger atypical cells are occasionally detected (11,12). The mitotic activity of the tumor cells varies, but abnormal mitotic figures are typically absent. Necrosis is rare, and is usually a feature associated with high-grade malignancies. The stroma may be variably collagenous or focally myxoid, and contain small numbers of lymphocytes, or on rare occasions, osteoclast-like giant cells. In addition, polygonal cells are occasionally observed in cellular areas (11,12).

LGMS has been reported at a variety of sites, including the extremities (17,18), trunk (19,20) and abdominal and pelvic cavities (21,22). However, the malignancy is usually associated with the head and neck, particularly the tongue (15). The present study investigated two rare cases of maxillary LGMS, one of which was misdiagnosed as an inflammatory myofibroblastic tumor (IMT) during a pre-operative excision biopsy, and presented with a different immunophenotype upon recurrence. In addition, the immunohistochemical analysis, differential diagnoses and literature of LGMS are described. This study was approved by the ethics commitee of (Jilin University Facilitated Oral Hospital, Changchun, China) and written informed consent was obtained from all patients.

## Materials and methods

### Tissues and reagents

The LGMS cases were retrieved from the routine surgical files at the Department of Pathology, Jilin University Facilitated Oral Hospital. Immunohistochemical analyses, using the primary antibodies listed in Table I, were performed upon 3-μm thick sections of paraffin-embedded, formalin-fixed tissue selected from each case. The monoclonal mouse anti-human primary antibodies against α-smooth muscle actin (SMA; 1:50), muscle-specific actin (MSA; 1:50), desmin (1:100), vimentin (1:100), h-caldesmon (1:50), cytokeratin (CK; 1:100), cluster of differentiation 34 (CD34; 1:100), anaplastic lymphoma kinase (ALK; 1:50), epithelial membrane antigen (EMA; 1:100) and Ki-67 (1:100), polyclonal rabbit anti-human antibody against fibronectin (1:100) and monoclonal rabbit anti-human against calponin (1:50) and S-100 protein (1:100) were purchased from Beijing ZhongShan Golden Bridge Biotechnology Co., Ltd. (Beijing, China). The immunohistochemical analysis was performed using the streptavidin-biotin-peroxidase complex method. Staining was scored according to the following criteria: −, <5% cells positive; +, 5–25% cells positive; ++, 25–75% cells positive; or +++, >75% cells positive.

### Case report

#### Case 1

In March 2010, a 45-year-old male, who presented with a painless, progressively enlarging swelling of the left maxilla that had been apparent for six months, was admitted to the Jilin University Facilitated Oral Hospital. The patient complained of discomfort whilst chewing, and had a history of malignant gingival fibroma on the left maxilla, which had been diagnosed and treated with a subsequent local wide resection of the tumor three years previously. The patient reported smoking a packet of cigarettes every day for the last 20 years, and consuming 50 ml of alcohol per day for the last 10 years. The results of all laboratory tests upon admission were within normal limits, and no clinical evidence of lymphadenopathy was apparent.

An oral examination revealed swelling of the left maxilla, involving the buccal and palatal alveolar bones and the palate, and spreading from the incisor (buccal side) and incisive foramen (palatal side) to the distal aspect of the third molar. The overlying mucosa exhibited no ulceration or hyperemia. All adjacent, associated teeth demonstrated grade II mobility. A computed tomography (CT) scan revealed a tumor mass measuring 5.2×4.5 cm within the left maxilla, which had invaded the left maxillary sinus, the nasal cavity, the left hard palate and the alveolar bone, with a relatively clear margin. A left maxillectomy was performed without any subsequent therapy. Evidence of recurrence or metastasis was not observed during the 30-month follow-up period.

#### Case 2

In October 2012, a 29-year-old female was admitted to the Jilin University Facilitated Oral Hospital following an excision biopsy that had been performed and which had been diagnosed as IMT. The patient presented with a progressively enlarging swelling of the anterior portion of the maxilla, accompanied with increased mobility of the upper incisors and pain during occlusion. The patient reported smoking four cigarettes per day for the last six years prior to admittance to the hospital. In 2000, the patient underwent an appendectomy, followed by a cesarean section nine years later. The results of all laboratory tests upon admission were within normal limits, and no clinical evidence of lymphadenopathy was apparent.

**Figure 1:**
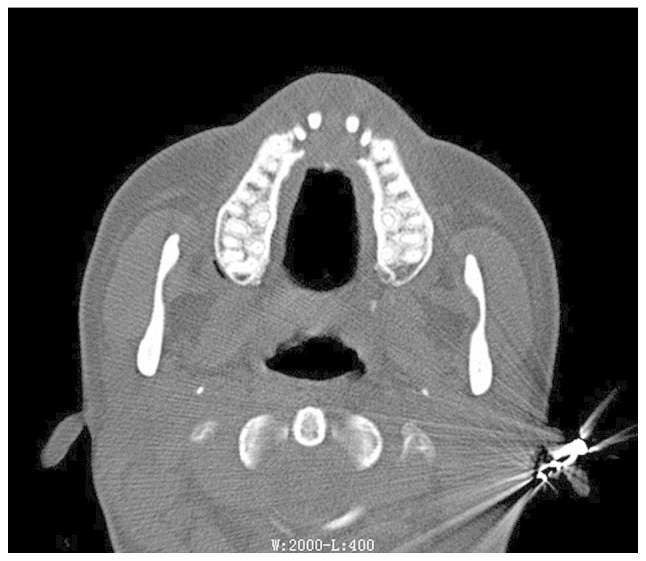
A computed tomography scan image revealing a low-grade myofibroblastic sarcoma. An oval-shaped swelling ~2.5×2.0 cm in size was observed in the anterior portion of the maxilla.

**Figure 2:**
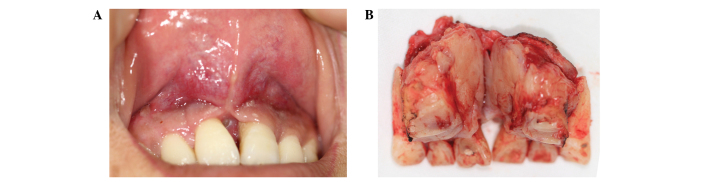
Macroscopic images revealing a low-grade myofibroblastic sarcoma in the maxilla (A) prior to and (B) following the surgery. (A) The overlying mucosa of the swelling demonstrating hyperemia. (B) The freshly-cut surface is pale yellow.

An oral examination revealed a stable, painful swelling in the anterior portion of the maxilla, involving the buccal and palatal alveolar bones, and extending from the distal aspect of the left maxillary incisor to the distal aspect of the right maxillary incisor. The overlying mucosa was hyperemic. A CT scan revealed a swelling ~2.5×2.0 cm in size, involving all maxillary incisors (Fig. 1). A wide local resection was performed from the distal aspect of one maxillary canine to that of the other (Fig. 2), and the tumor-free margins were histologically observed in the frozen sections obtained from the biopsies. Ancillary therapy was not used, and a diagnosis of LGMS was reached following surgery. However, six months later, the neoplasm recurred, this time measuring 1.0×1.5 cm in size. A CT scan revealed bone erosion on the buccal side of the first premolar in the right maxilla. A local wide resection was therefore performed, which included the removal of certain tumor-free margins. Further evidence of recurrence or metastasis was not observed at the 6-month follow-up.

## Results

### Case 1

**Figure 3:**
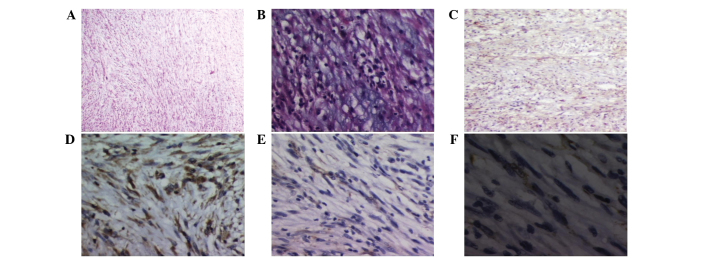
Micrographs revealing the histological appearance of the tumor in case 1. (A) Hematoxylin and eosin stain revealing spindle cells arranged in long fascicles (magnification, ×40). (B) Myxoid degeneration apparent in the stroma (magnification, ×200). (C) Spindle cells positive for fibronectin (magnification, ×100). (D) Spindle cells positive for vimentin (magnification, ×200). (E and F) Spindle cells positive for α-smooth muscle actin. Magnification, (E) ×200 and (F) ×400.

Microscopic examination revealed a tumor primarily composed of spindle-shaped cells, with diffuse infiltrative growth into the surrounding tissues (Fig. 3A). The neoplastic spindle cells were predominately arranged in long fascicles, while herringbone and vague storiform patterns were observed focally. The cytoplasm demonstrated a pale eosinophilic appearance with ill-defined cellular margins. The nuclei appeared to be either tapered and wavy, or slightly round with myxoid degeneration of the stroma (Fig. 3B). Nuclear atypia was mild with occasional nuclear hyperchromatism. Necrosis was not observed, and the mitotic rate was relatively low at 1–2/high-power field (HPF), with the absence of any abnormal patterns of mitosis. Inflammatory components were unremarkable in amount. The immunohistochemical analysis revealed that the spindle cells were positive for fibronectin (FN) (+) (Fig. 3C), vimentin (++) (Fig. 3D) and SMA (+) (Fig. 3E and F), but negative for S-100 (−).

### Case 2

**Figure 4:**
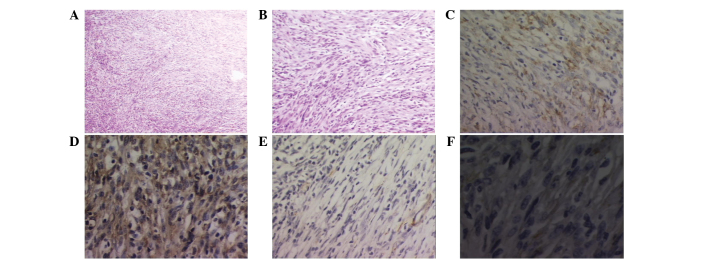
Micrographs revealing the histological appearance of the tumor in case 2. (A) Hematoxylin and eosin staining revealing spindle cells arranged in fascicles of varying length (magnification, ×40). (B) An inflammatory background is apparent in the stroma (magnification, ×100). (C) Spindle cells positive for desmin (magnification, ×200). (D) Spindle cells positive for vimentin (magnification, ×200). (E) Spindle cells positive for α-smooth muscle actin (magnification, ×200). (F) Spindle cells positive for fibronectin (magnification, ×400).

Histological examination revealed a tumor primarily composed of spindle-shaped cells arranged in fascicles of varying length, with intersecting and storiform patterns apparent focally (Fig. 4A). The spindle cells had ill-defined, palely eosinophilic cytoplasm, and the nuclei were either thin and wavy or slightly round and vesicular. Nuclear atypia was mild, while nuclear hyperchromatism was occasionally observed. The mitotic rate was 1–2/HPF and abnormal mitotic patterns were not observed. A diffuse inflammatory infiltration (Fig. 4B), comprised of a large number of lymphocytes and occasional plasma cells, was also identified. The hypocellular portion of the stroma presented with hyaline degeneration.

**Figure 5:**
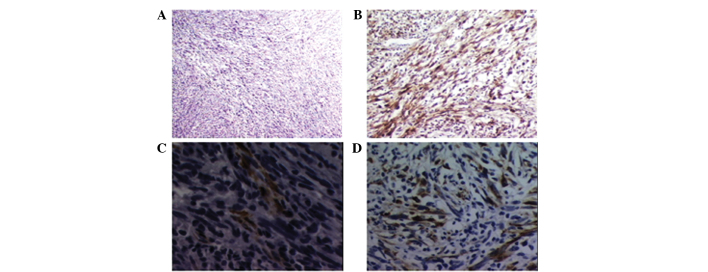
Micrographs revealing the histological appearance of the recurrent lesion in case 2. (A) Hematoxylin and eosin staining revealing spindle cells arranged in fascicles (magnification, ×40). (B) Spindle cells positive for fibronectin (magnification, ×100). (C) Spindle cells positive for α-smooth muscle actin (magnification, ×400). (D) Spindle cells positive for cytokeratin (magnification, ×200).

The immunochemical analysis revealed that the spindle cells were positive for desmin (+) (Fig. 4C), vimentin (+++) (Fig. 4D), α-SMA (+) (Fig. 4E) and FN (Fig. 4F), and negative for S-100 protein (−). The recurrent tumor demonstrated no further malignancy (Fig. 5A), but presented with a different immunophenotype, which was positive for FN (++) (Fig. 5B), SMA (+) (Fig. 5C) and CK (+) (Fig. 5D) and negative for desmin (−). The diagnosis was consistent with the primary lesion. The primary antibodies used in the present study, and the results of the immunohistochemical analysis, are summarized in Table I.

## Discussion

The term myofibrosarcoma, which defines a malignant tumor of the myofibroblasts, was suggested by Ghadially (23) in 1980 as an analogy of the term fibrosarcoma. According to current grading systems, myofibroblastic sarcomas (myofibrosarcomas) can be classified into low-, intermediate- and high-grade entities. Pleomorphic myofibroblastic sarcoma is a high-grade subset of the malignant fibrous histocytoma, and can be distinguished from the morphologically similar low- and intermediate-grade myofibroblastic sarcomas. The majority of LGMSs are deep soft-tissue lesions, but others may be intraosseous or have originated from subcutaneous tissue or the submucosa. The age range of patients with LGMS is 4–85 years (mean, 40 years), with a slightly higher proportion of males affected. The tumors range in size between 1.5 and 17 cm (24). By contrast, myofibroblastic sarcomas of the mammary gland differ clinically and biologically from those affecting extra-mammary regions. The aggressive mammary myofibroblastic sarcomas predominantly affect middle- to old-aged patients (46 to 81 years), and are more frequently observed in females (25). A study by Yamada *et al* (26), reported a 38.2% recurrence rate among 38 cases of LGMS, which is one of the highest values cited from nasal cavity/paranasal sinus LGMSs, the second highest after cases of the jawbone, followed by the deep tissue space. The recurrence rate for tumors of <3 cm in size is 21.4%, but for tumors >3 cm, the recurrence rate increases to 46.2% (26). In the aforementioned study, the common sites affected by LGMS, after the tongue, are the maxilla and palate, the mandible, the nasal/paranasal cavity and the deep tissue spaces, including the parapharyngeal space.

Common factors associated with tumor recurrence are believed to be tumor size, growth pattern and location, and the surgical methods used during treatment. However, it is yet to be demonstrated whether the immunophenotype of LGMS is a contributing factor to recurrence. Myofibroblastic sarcomas, except for pleomorphic types, are slow-growing, infiltrative tumors that are susceptible to local recurrence, but rarely metastasize, even after a number of years. A painless, enlarging mass is the most common clinical finding, but tumor-related symptoms, such as hoarseness (27) and dysphonia (28) in cases of the larynx, and paresthesia (29,30), ulceration and non-allergic to topical steroids (31), are also reported. In addition, lung metastasis was observed in one case in each of the large series reported by Mentzel *et al* (15) and Montgomery *et al* (32), and has also been identified in other published studies (25,33,34).

Although LGMSs can be focally contained, the majority infiltrate into the adjacent fibrous tissue, fat, skeletal muscle or bone. Recurrent tumors tend to exhibit increased pleomorphism, or display areas of higher grade malignancy (32,33), but a pulmonary metastasis from an intermediate-grade myofibrosarcoma that was unremarkable and fasciitis-like has also been reported (32,33).

LGMSs display variable immunophenotypes, despite the morphological homogeneity. In addition to being positive for vimentin, these sarcomas may be positive for either SMA and/or desmin (15,32,33), resulting in desmin(+)/SMA(−), desmin(−)/SMA(+) and desmin(+)/SMA(+) immunophenotypes. Reflecting the microanatomy of myofibroblasts, SMA staining can be present as a peripheral rim beneath the cell membrane. Calponin is diffusely positive in the majority of cases, while h-caldemon is usually negative. Myofibroblastic sarcomas can also display fibronectin (35), but not collagen IV or laminin. EMA and S-100 protein staining is almost always negative, whereas keratin(+) and CD34(+) cells have occasionally been reported (15). Electron microscopy has revealed that the tumor cells exhibit features of myofibroblasts, but with variable numbers of fibronexuses that tend to be modestly developed in comparison with those observed in granulation tissue and tumor stroma (36,37). Cytogenetic analyses presented by Mentzel *et al* (15) and Meng *et al* (38) demonstrated non-specific findings, such as those of infantile fibrosarcama and inflammatory myofibroblastic sarcoma.

The tumor cells from the two cases in the present study did not express calponin or MSA, a finding which is not in accordance with the reported literature. Despite this, the other results obtained from the present study are supportive of a diagnosis of LGMS. The lesion from case 2, which was misdiagnosed as an IMT following a pre-operative excision biopsy, presented with a different immunophenotype upon recurrence. In contrast to the primary lesion, tumor cells of the recurrent lesion were negative for desmin and positive for CK. This is a rare finding that has not been previously reported. On the basis of this transformation, the present study concluded that cases of LGMS with immunoprofile alterations are predictive of relatively poor prognoses, particularly in terms of recurrence rate. As previously stated, recurrent LGMS tends to exhibit increased pleomorphism and areas of higher grade malignancy. Although the recurrent tumor in case 2 did not exhibit such alterations, it is hypothesized that further recurrent lesions would potentially present with increased pleomorphism and aggressiveness. However, the evaluation and follow-up of additional cases is required in order to understand this phenomenon.

A wide local resection, including the removal of tumor-free margins, is the primary treatment for LGMS. However, ancillary treatments, such as chemotherapy and radiotherapy, are of uncertain clinical value as there have been reported cases whereby LGMS, excised along with free margins, demonstrated no recurrence or metastasis either with or without ancillary treatment (39–42). Furthermore, certain tumors have recurred or metastasized subsequent to the administration of ancillary treatment (25). A previous study also reported that unresectable LGMS demonstrated no response to the combination of chemotherapy and radiotherapy, and resulted in mortality (28). In the present study, the patients underwent wide local tumor excision, which included the removal of tumor-free margins, and did not receive further ancillary therapy. The lesion in case 2 recurred six months later, and a wide local excision was performed again. Even though no further recurrence or metastasis was identified six months post-operatively, further follow-up is imperative.

Myofibroblastic sarcoma must be distinguished from other neoplasms, as the lesion can resemble varying types of benign myofibroblastic tumors, including nodular fasciitis, proliferative myositis and fibromatosis, and also other types of low-grade spindle-cell sarcomas, notably leiomyosarcoma and fibrosarcoma. The differential diagnosis is usually made based upon clinical and morphological features, combined with immunohistochemical analysis and occasionally ultrastructural features (11,12). As case 2 in the present study was misdiagnosed as an IMT, a description of the differential diagnosis is subsequently provided.

According to the 2002 World Health Organization classification, IMT, which is synonymous with inflammatory fibrosarcoma (43), is a locally aggressive and rarely metastatic intermediate tumor (44). The tumor usually arises within the lungs or the abdomen, particularly within the retroperitoneum or mesentery, and on occasions in the visceral and soft-tissue sites of the head, neck, trunk and extremities. IMT forms a solitary or multicentric mass up to ~10 cm in diameter, has a peak incidence in childhood or adolescence and exhibits a female predominance (45,46). Furthermore, systemic features, including anemia and hypergammaglobulinemia, which are potentially associated with interleukin-6, are occasionally present. All the aforementioned clinical features differ from those of LGMS.

Despite the similar infiltrating capacities of IMT and LGMS, the histological growth patterns of the two lesions are generally different. IMT presents with fasciitis-like, fascicular and fibrosarcoma- or leiomyosarcoma-like hypocellular fibrous areas, regions of hyalinization and calcification, and is accompanied by permanent inflammation in the stroma, which is primarily comprised of polyclonal plasma cells and lymphocytes (24). The incidence of pleomorphism or necrosis is low. Histologically, IMT is multifocally- or diffusely-positive for α-SMA and MSA, and occasionally for desmin. Certain CKs are also evident in certain cases, particularly in intra-abdominal tumors (24). The immunoreactivity profile of ALK reflects rearrangements in the ALK gene, which is located on chromosome 2p23 and encodes a tyrosine kinase receptor. A variety of fusion partners exist, of which tropomyosin 3 and 4 are the most common (47).

When LGMS is accompanied by an inflammatory background, it is increasingly difficult to distinguish it from IMT. This often results in misdiagnosis, as in case 2 in the present study. Occasionally, spindle-cell sarcoma, monophasic synovial sarcoma, malignant peripheral nerve sheath tumors and certain cases of angiosarcoma and spindle-cell rhabdomyosarcoma are involved in the differential diagnosis.

Although the proportion of neoplastic myofibroblasts required for a diagnosis of LGMS is not defined, the disease is widely accepted as a distinct entity. Despite the fact that it exhibits improved diagnostic specificity compared with myeloid markers, the fibronexus has also been identified in fetal endothelial cells, spindle-cell carcinomas (48) and more rarely in neoplastic, compared with non-neoplastic, myofibroblasts. In recent years, the ultrastructural features of myofibroblasts have been complemented by histological and immunohistochemical criteria to produce a comprehensive definition. Therefore, cases of LGMS that exhibit actin and fibronectin positivity, but h-caldesmon and smooth muscle myosin negativity, will for the majority of pathologists without access to electron microscopy, be sufficient to reach a diagnosis. However, in circumstances where it may be challenging to interpret the results, for example, in instances of focal or equivocal immunostaining, electron microscopy would be a useful tool to provide maximum diagnostic confidence.

Although genetic changes have been identified that prove that LGMS is a definite neoplasm, specific changes, such as those evident in cases of infantile fibrosarcoma and IMT, require identification by the application of suitable techniques to better describe this entity.

## Figures and Tables

**Table I:** Immunohistochemical antibodies and results.

			Immunoreactivity		
			
Antibody	Clone	Pretreatment	Case 1	Case 2 (primary lesion)	Case 2 (recurrent lesion)
Vimentin	V9	None	+	+	+
Desmin	ZC18	None	−	+	−
SMA	1A4	None	+	+	+
MSA	HHF35	None	−	−	−
EMA	E29	Pressure cooker	−	−	−
CK	AE1/AE3	Pressure cooker	−	−	+
ALK	ALK-1	Pressure cooker	−	−	−
Ki-67, %	K-2	Pressure cooker	24 (20–27)	24.7 (20–27)	41.9 (34–49)
S-100	4C4.9	Pressure cooker	−	−	−
CD34	QBEnd/10	Pressure cooker	−	−	−
Calponin	EP63	Pressure cooker	−	−	−
h-caldesmon	h-CD	Pressure cooker	−	−	−
Fibronectin	polyclone	Pressure cooker	+	+	+

SMA, smooth muscle antigen; MSA, muscle-specific actin; EMA, epithelial membrane antigen; CK, cytokeratin; ALK, anaplastic lymphoma kinase; CD34, cluster of differentiation 34. Data for Ki-67 are presented as mean (range) percentage values.
